# Routinely collected antenatal data for longitudinal prediction of preeclampsia in nulliparous women: a population-based study

**DOI:** 10.1038/s41598-021-97465-3

**Published:** 2021-09-09

**Authors:** Anna Sandström, Jonathan M. Snowden, Matteo Bottai, Olof Stephansson, Anna-Karin Wikström

**Affiliations:** 1 Department of Women’s and Children’s Health, Uppsala University, Uppsala, Sweden; 2 Clinical Epidemiology Division, Department of Medicine Solna, Karolinska Institutet, Stockholm, Sweden; 3 Department of Women’s Health, Karolinska University Hospital, Stockholm, Sweden; 4 Department of Obstetrics and Gynecology, Oregon Health and Science University, Portland, OR USA; 5 School of Public Health, Oregon Health and Science University-Portland State University, Portland, OR USA; 6 Division of Biostatistics, Institute of Environmental Medicine, Karolinska Institutet, Stockholm, Sweden; 7 Department of Medicine Solna, Karolinska Institutet, Clinical Epidemiology Division T2, Karolinska University Hospital, 171 76 Stockholm, Sweden

**Keywords:** Pre-eclampsia, Epidemiology, Risk factors

## Abstract

The objective was to evaluate the sequentially updated predictive capacity for preeclampsia during pregnancy, using multivariable longitudinal models including data from antenatal care. This population-based cohort study in the Stockholm-Gotland Counties, Sweden, included 58,899 pregnancies of nulliparous women 2008–2013. Prospectively collected data from each antenatal care visit was used, including maternal characteristics, reproductive and medical history, and repeated measurements of blood pressure, weight, symphysis-fundal height, proteinuria, hemoglobin and blood glucose levels. We used a shared-effects joint longitudinal model including all available information up until a given gestational length (week 24, 28, 32, 34 and 36), to update preeclampsia prediction sequentially. Outcome measures were prediction of preeclampsia, preeclampsia with delivery < 37, and preeclampsia with delivery ≥ 37 weeks’ gestation. The area under the curve (AUC) increased with gestational length. AUC for preeclampsia with delivery < 37 weeks’ gestation was 0.73 (95% CI 0.68–0.79) at week 24, and increased to 0.87 (95% CI 0.84–0.90) in week 34. For preeclampsia with delivery ≥ 37 weeks’ gestation, the AUC in week 24 was 0.65 (95% CI 0.63–0.68), but increased to 0.79 (95% CI 0.78–0.80) in week 36. The addition of routinely collected clinical measurements throughout pregnancy improve preeclampsia prediction and may be useful to individualize antenatal care.

## Introduction

As a complex multifactorial disorder affecting 3–5% of pregnancies, preeclampsia remains a leading cause of maternal and perinatal mortality and morbidity worldwide, and the incidence is increasing^1–4^. Aspirin prophylaxis from early pregnancy to a defined high-risk population is effective for the prevention of *preterm* preeclampsia (delivery < 37 weeks’ gestation)^5^, possibly also when treatment is initiated after 16 weeks’ gestation^6^. Preeclampsia symptoms can develop rapidly and early detection of preeclampsia is crucial for appropriate antenatal and perinatal surveillance and medical care^1,7,8^. Therefore, it is critical that research continues the search for effective, safe, and affordable screening methods for preeclampsia, not only conducted in first trimester, but also throughout pregnancy.

In the last decade, advanced predictive multivariable *early* pregnancy models for preeclampsia have achieved good risk discrimination for *preterm* preeclampsia^9,10^. Further studies are yet warranted to establish an implementable model or models for widespread use in clinical practice. This also includes the cost-effectiveness of using non-routinely performed examinations^4,11–13^. Crucially, prediction of the dominating *term* preeclampsia (delivery ≥ 37 weeks’ gestation) remains elusive, and the predictive accuracy declines as gestational age of onset increases^14,15^. A short interval between screening and preeclampsia onset, and including serial measurements of predictors, has been shown to improve the prediction^16–18^.

Previous knowledge implies that patterns of blood pressure, hemoglobin, weight gain and symphysis-fundal height throughout pregnancy, as well as hyperglycemia and isolated proteinuria may represent useful markers for the risk of preeclampsia, possibly improving with gestational age and if combined in multivariable models^13,19–33^. Routinely collected clinical data that changes dynamically throughout pregnancy as blood pressure, maternal weight, blood glucose, symphysis-fundal height, proteinuria, hemoglobin and blood sugar levels are widely available. Yet few studies to date have taken advantage of this feature of data in multivariable analysis, to extract valuable inherent and interacting information^18,34^. The analytical and computational tools needed to enable such analysis are substantial. Further, nulliparous women have higher risk of preeclampsia and no marker of risk based on previous obstetric history, and the predictive capacity in multivariable models is lower among nulliparous than parous women^9,35–37^. Our hypothesis was that routinely collected early pregnancy and antenatal care data throughout pregnancy could improve prediction of preeclampsia when evaluated in a multivariable fashion, with updated prediction at each visit.

In this population-based cohort study of 58 899 nulliparous women we included 20 early pregnancy variables and seven longitudinal repeatedly collected variables relevant for the prediction of preeclampsia. Using millions of data points, we created a shared-effect joint longitudinal model using all available information up until a given visit, irrespective of varying number and timing of visits, with the objective to iteratively update preeclampsia prediction over time.

## Methods

### Setting and data sources

The population-based Stockholm-Gotland Obstetric Database includes electronically transferred data from computerized antenatal care, delivery and postnatal records from all units in the Stockholm-Gotland area where approximately one fourth of all births in Sweden occur^38^. The detailed, standardized, prospectively collected data include demographic, medical and reproductive history, and information on serial examinations from each visit to the attending midwife or physician in antenatal care.

The pregnancies in the database were individually linked to the National Patient Register^39^, including diagnoses on inpatient admissions and hospital outpatient visits according to the Swedish version of International Classification of Diseases (ICD) 10th revision. Linkage to the Swedish Prescribed Drug Register^40^, holding data on all prescribed substances, ATC-code (Anatomical Therapeutic Chemical classification) and date of purchase, for all dispensed drugs in the outpatient population was conducted.

### Study population

Live-born singleton births between January 1st, 2008 and December 31st, 2013 were included in the study base (n = 149,298). Births from gestational week 22 of nulliparous women were included (n = 68,928). Pregnancies without information on gestational length, or with major congenital anomaly (defined as any major malformation according to the register of birth defects^41^) diagnosed in the National Patient Register were excluded, resulting in a study population of 58,899 singleton pregnancies. We conducted sensitivity analyses where pregnancies with maternal use of aspirin during pregnancy were excluded (n = 623), since this can alter the performance of the predictive models. Use of aspirin during pregnancy was defined as purchased prescription of aspirin during pregnancy in the Swedish Prescribed Drug Register (a prescription is needed for aspirin of doses indicated during pregnancy).

### Study variables

Gestational length was determined using the following hierarchy: (a) date of embryo transfer, (b) first or early second trimester ultrasound, (c) date of last menstrual period, and (d) postnatal assessment. At the first visit to antenatal care, around gestational week 10, the woman is interviewed regarding demographic features, maternal reproductive and medical history, and the information is registered in the antenatal record (defined as baseline variables in our study). During pregnancy, women usually have additional 10–12 visits, where data are collected and recorded by midwives or physicians in a standardized way (defined as longitudinal variables in our study). At the first and each following visit, one or several medical examinations are performed. The data from each visit throughout pregnancy, collected in antenatal records, compose the predictive variables in the models for preeclampsia in the study.

#### Baseline variables

All the variables from first antenatal visit were treated as continuous or categorized as presented in Table 1. The following baseline variables were included in the multivariable model: Self-reported information on region of birth, family situation, height, smoking habits three months before and in early pregnancy, reproductive history (previous miscarriage, infertility duration, assisted reproduction), family history of preeclampsia and hypertension, and medical history was further collected**.** Pre-existing diabetes included diabetes type I and II. The collected information was registered in a standardized way either as tick boxes, pre-specified options, or as numbers. Family history of hypertensive diseases was however registered as free text, and based on this, two dichotomous variables (family history of hypertension and family history of preeclampsia) were constructed. Venous sampling for blood group was routinely conducted in early pregnancy.

**Table 1:** Predictive variables routinely collected at first antenatal visit in the study population of 58 899 nulliparous women.

Predictive variables collected at first antenatal visit	N	Without preeclampsia n = 56,323		With preeclampsia n = 2576		P-value
		N		N		
Maternal age, years*			29.3 (5.0)		29.9 (5.3)	< 0.001
**Region of birth n, %**						< 0.001
Sweden		41,778	75.2	2021	80.1	
Nordic countries (except of Sweden)		896	1.6	39	1.5	
Europe (except of Nordic countries)		4235	7.6	144	5.7	
Africa		1759	3.2	101	4.0	
North America		368	0.7	13	0.5	
South America		951	1.7	33	1.3	
Asia		5544	10.0	170	6.7	
Oceania		50	0.1	3	0.1	
Missing n	794					
**Family situation n, %**						0.16
Single		1159	2.1	67	2.6	
Living with partner		51,781	92.5	2354	92.1	
Other		3028	5.4	136	5.3	
Missing n	374					
Height, cm*		166.6	6.5	166.1	6.5	< 0.001
Missing n	395					
**Smoking 3 months before pregnancy n, %**						0.38
< 10		4881	8.7	220	8.6	
≥ 10		4093	7.3	169	6.6	
Missing n	365					
**Smoking at registration n, %**						0.082
< 10		1952	3.5	70	2.7	
≥ 10		353	0.6	20	0.8	
Missing n	333					
**Previous miscarriage n, %**		9924	17.6	486	18.9	0.10
**Infertility duration, years, %**						< 0.001
1–2		6223	11.0	300	11.6	
> 2		3016	5.4	201	7.8	
**Infertility treatment n, %**						0.009
Ovarian stimulation		816	1.4	44	1.7	
IVF		3717	6.6	207	8.0	
**Family history of preeclampsia n, %**		140	0.2	16	0.6	< 0.001
**Family history of hypertension n, %**		9358	16.6	593	23.0	< 0.001
**Cardiovascular disease n, %**		732	1.3	46	1.8	0.035
**Endocrine disease n, %**		2770	4.9	164	6.4	< 0.001
**Pre-existing diabetes n, %**		247	0.4	59	2.3	< 0.001
**Thrombosis history n, %**						
**SLE n, %**		61	0.1	3	0.1	0.90
**Chronic hypertension n, %**		247	0.4	40	1.6	< 0.001
**Mb Crohn/Ulcerative colitis n, %**		478	0.8	17	0.7	0.30
**Chronic kidney disease n, %**		249	0.4	26	1.0	< 0.001
**Blood group n, %**						0.15
0		19,828	38.1	869	36.8	
A		22,385	43.1	1034	43.8	
AB		2881	5.5	117	5.0	
B		6897	13.3	342	14.5	
Missing n	4546					

*Mean (SD).

#### Time-varying variables

Our study is distinguished by inclusion of repeated measures of time-varying physiological parameters in the multivariable model. These included repeated examinations of systolic and diastolic blood pressure, maternal weight, hemoglobin and capillary glucose levels, urine dipstick for protein, and from gestational week 24, measurements of symphysis-fundal height. The number of observations and time-points differed between all women. The longitudinal predictors were treated as continuous except for plasma glucose and proteinuria, which were categorized as below. Maternal blood pressure was measured by the midwife in supine position on the right upper arm using manual blood pressure equipment with a cuff size appropriate for arm circumference. Korotkoff V was used for diastolic blood pressure. Weight was measured by the midwife. Capillary blood sampling was conducted for plasma glucose routinely during pregnancy on all women, categorized as dichotomous, normal or high (≥ 9 mmol/L), and for hemoglobin level. Urine dipstick tests for protein were collected and categorized as 0, 1, or ≥ 2.

#### Outcome variable

Diagnosis of preeclampsia was classified according to the Swedish version of ICD 10th codes (O14.0, O14.1, O14.9 or O15), by the responsible doctor during pregnancy or at discharge, and was retrieved from the National Patient Register at either; (1) an inpatient admission or, (2) an outpatient visit followed by either a second outpatient visit or an inpatient admission, where the date of the first diagnosis was used. Preeclampsia was during this time-period defined as hypertension (blood pressure ≥ 140 mmHg and/or diastolic blood pressure ≥ 90 mmHg two times with at least an interval of 4 h), combined with proteinuria (≥ 0.3 g/24 h) occurring after 20 weeks’ gestation, or as superimposed preeclampsia, chronic hypertension with addition of proteinuria.

#### Predicted outcomes

The main outcomes were defined as diagnosis of preeclampsia, and were categorized as: (1) preeclampsia: any time in pregnancy; (2) *preterm* preeclampsia: diagnosis and delivery < 37 weeks’; and (3) *term* preeclampsia: diagnosis before or from 37 weeks with delivery ≥ 37 weeks’ gestation. We were also interested in the timing of preeclampsia *diagnosis*. Therefore, secondary outcomes were defined as diagnosis of preeclampsia: (4) < 37 weeks’ gestation and (5) ≥ 37 weeks’ gestation (irrespective of gestational age at delivery).

### Statistical methods

We utilized a modelling approach that builds on joint models, a method for modelling time-to-event outcomes in a survival analysis framework^42^. We sought to conduct an iteratively updated prediction process (e.g., at 24 weeks, again at 28 weeks, etc.), so we further utilized a shared-effects approach to build joint longitudinal models. This shared-effects joint longitudinal model utilizes all of the longitudinal measurements on each woman, irrespective of timing and number of observations. The predictive models included the twenty early pregnancy predictors (Table 1), the two categorical time-varying predictors (Table 2), and information from the five continuous time-varying predictors (systolic and diastolic blood pressure, maternal weight, hemoglobin and capillary glucose levels, proteinuria, and symphysis-fundal height). These models were built in three subsequent steps that are detailly described in Appendix S1 in Data Supplement. Briefly:

1. The average trajectory of the five different time-varying predictors in the population without preeclampsia were calculated by a mixed effect model.
2. The approximation of the actual trajectories of each of the five time-varying predictors for each woman (with and without preeclampsia) was captured by the main features of (a) level, (b) trend, and (c) curvature (Fig. S1). The departure of the woman’s trajectory from the non-preeclamptic population’s mean trajectory is represented by the standard deviations, u-scores (similar to z-scores), for each of the three features, for each of the five time-varying predictors.
3. We estimated a generalized linear model for each preeclampsia outcome above, using the baseline predictors, the binary glucose and categorical proteinuria predictors, and the three u-scores for level, trend and curvature for each of the five longitudinal variables.

**Table 2:** The predictive variables of capillary glucose and proteinuria, collected at repeated time points in antenatal care in the study population of 58 899 nulliparous women.

Predictive variables at first antenatal visit	Without preeclampsia n = 56,323		With preeclampsia n = 2576		P-value
	N		N		
Capillary glucose ≥ 9 mmol/L* %	1 842	3.3	135	5.3	< 0.001
Proteinuria dipstick^†^ %					< 0.001
1+	5 779	10.5	666	26.0	
≥ 2+	745	1.4	1 140	44.4	

*Dichotomous time-varying predictor: if ≥ 9 mmol/L at any antenatal visit, then positive.

^†^Categorical time-varying predictor, if 1+ or ≥ 2+, at any antenatal visit, otherwise 0.

There was not a substantial proportion of missing data among the twenty early pregnancy predictors, nor from the time-varying predictive variables were all observations were used and therefore no specific missing data analysis was made.

#### Assessment of the performance of the predictive models

Accuracy of prediction of the outcomes with these models were possible to evaluate at any given gestational age, only including women not yet affected by preeclampsia. The predictive capacity of the models in completed gestational weeks of 24, 28, 32, 34 and 36 were performed and quantified by area under the curve (AUC), with 95% confidence intervals (CI), and by detection rates (i.e. sensitivity) for 10% false positive rates.

Statistical analyses were done with Stata 15 (StataCorp, College Station, TX, USA) The study is presented according to the TRIPOD guidelines^43^.

This study was approved by the regional ethical committee (IRB) at Karolinska Institutet, Stockholm, Sweden 02/04/2009 no 2009/275-31, and 24/02/2012 no 2012/365-32. The studies in the project are based on previously collected medical record and register data and the personal identification numbers has been replaced by anonymous serial numbers by the Swedish National Board of Health and Welfare. Analyses were conducted on de-identified data and no informed consent was needed according to the ethical approvals approved by the ethical committee at Karolinska Institutet, Stockholm, Sweden (no 2009/275-31 and no 2012/365-32). All methods were performed in accordance with relevant guidelines and regulations.

## Results

In the study population of 58 899 nulliparous women, 2 576 (4.4%) developed preeclampsia during pregnancy. Aspirin was used by 623 (1.1%) of the women. Demographic, reproductive and medical history variables from first antenatal visit are presented in Table 1, stratified by women without and with preeclampsia. Women who developed preeclampsia were slightly older, more often born in Sweden or Africa, of shorter height, more often having longer infertility duration and assisted reproduction, compared to women who did not develop preeclampsia. Family history of preeclampsia and hypertension, and chronic diseases were more common among women who developed preeclampsia (Table 1).

**Figure 1:**
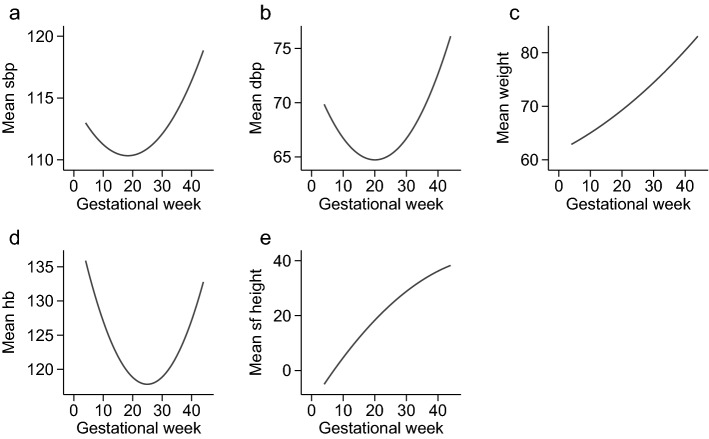
Among women not developing preeclampsia, trajectories of: (**a**) mean systolic blood pressure (sbp), (**b**) mean diastolic blood pressure (dbp), (**c**) mean maternal weight (weight), (**d**) mean symphysis-fundal measure (fundus), and (**e**) mean haemoglobin level (Hb) throughout pregnancy.

The median number of visits in antenatal care was 11, and 12 among women without and with preeclampsia, respectively (Table S1). Table 2 present the categorical time-varying variables. Capillary glucose ≥ 9 mmol/L and proteinuria in antenatal care were both more common among women who later developed preeclampsia. Figure 1 displays the trajectories of the additional five time-varying variables in the non-preeclamptic population. Systolic and diastolic blood pressure levels as well as hemoglobin level all have a prominent decrease in second trimester (Fig. 1).

For the time-varying variables, a set of three u-scores captured the departure (level, trend and curvature) of each woman’s trajectory from the non-preeclamptic population. The mean u-scores for level, trend and curvature for the non-preeclamptic and preeclamptic population, respectively, are presented in Table S2. The mean u-scores for the curvatures of systolic and diastolic blood pressure, and maternal weight were largely increased in women with preeclampsia compared to women without preeclampsia (Table S2).

**Figure 2:**
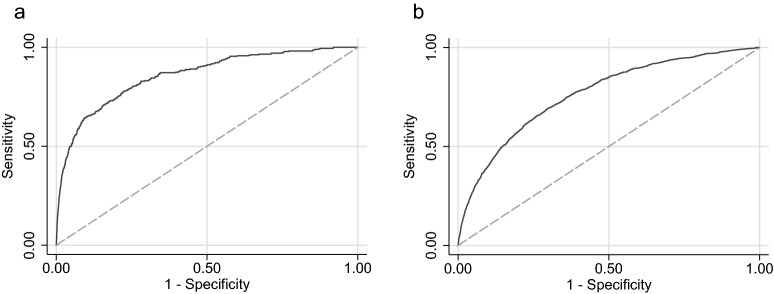
ROC curves for prediction of (**a**) *preterm* preeclampsia at 34 weeks’ gestation and (**b**) prediction of *term* preeclampsia at 36 weeks’ gestation.

The ability to predict the three groups of preeclampsia at the time-points gestational week 24, 28, 32, 34 and 36 are presented as AUC with 95% CI, as sensitivity at a fixed false positive rate of 10% with 95% CI (Table 3), and as ROC-curves (Fig. 2). The AUC and sensitivity generally increased with gestational length at prediction for all three groups of preeclampsia (Table 3). For women with preeclampsia with delivery ≥ 37 gestational weeks, the predictive capacity in gestational week 24 was lower compared to preeclampsia with delivery < 37 weeks, but increased to 0.79 (95% CI 0.78–0.80) in gestational week 36 (Table 3). The predictive capacity for *diagnosis* of preeclampsia < 37 weeks and ≥ 37 weeks (irrespective of gestational age at delivery), generally showed similar results and are presented in Table S3. In sensitivity analyses of women without aspirin treatment, the results were similar as in the analysis of the entire study population (Table S4). Table S5 displays the coefficients of the parameters of the predictive model and Table S6 displays the parameters of the mixed effect model.

**Table 3:** Performance of the predictive models for preeclampsia, preeclampsia with delivery < 37 weeks and preeclampsia with delivery ≥ 37 weeks at different gestational ages in the in the study population of 58 899 nulliparous women.

Gestational age of prediction^†^ (weeks)	Preeclampsia				Preeclampsia with delivery < 37 weeks’ gestation				Preeclampsia with delivery ≥ 37 weeks’ gestation*			
	AUC^‡^	(95% CI)	Sensitivity for 10% FPR^§^	(95% CI)	AUC^‡^	(95% CI)	Sensitivity for 10% FPR^§^	(95% CI)	AUC^‡^	(95% CI)	Sensitivity for 10% FPR^§^	(95% CI)
24	0.69	(0.66–0.71)	29.0	(25.5–34.0)	0.73	(0.68–0.79)	37.2	(26.5–48.9)	0.65	(0.63–0.68)	25.2	(20.9–29.9)
28	0.70	(0.69–0.72)	30.2	(29.5–33.6)	0.78	(0.76–0.81)	43.0	(37.2–48.9)	0.67	(0.66–0.69)	25.5	(23.5–27.6)
32	0.73	(0.72–0.75)	35.4	(33.3–37.5)	0.85	(0.82–0.87)	59.7	(52.9–66.3)	0.71	(0.69–0.72)	29.6	(27.4–31.7)
34	0.77	(0.75–0.78)	41.0	(38.8–43.3)	0.87	(0.84–0.90)	66.4	(57.6–74.4)	0.75	(0.73–0.76)	37.5	(35.2–39.8)
36	0.80	(0.78–0.81)	46.4	(43.9–48.8)	0.84	(0.77–0.92)	50.0	(29.1–70.9)	0.79	(0.78–0.80)	45.0	(42.6–47.5)

*Diagnosis of preeclampsia at any gestational length with delivery ≥ 37 weeks.

^†^The model is composed of the predictive variables collected at first antenatal visit, the time-varying predictors plasma glucose and proteinuria, and the u-scores of level, trend and curvature for each of the time-varying predictors systolic and diastolic blood pressure, haemoglobin, maternal weigh and symphysis fundal height up until the gestational week of prediction (24, 28, 32, 34 and 36).

^‡^AUC: Area under receiver operating characteristic curve.

^§^FPR: False positive rate.

## Discussion

### Main findings

This is the first longitudinal preeclampsia prediction study of nulliparous women taking early pregnancy predictors together with several clinical routinely collected examinations with serial measurements throughout pregnancy. The predictive accuracy of the models for *preterm* and *term* preeclampsia generally increased with gestational age at time of prediction from gestational week 24 and onwards. Our findings demonstrate the importance of using clinical information during pregnancy for risk evaluation of preeclampsia.

### Interpretation

In the SCOPE study, a predictive model for preeclampsia in nulliparous women based on maternal clinical predictors including MAP at 15 weeks’ gestation, AUC under internal validation was 0.71^36^. This was similar to our findings in nulliparous women with an AUC of 0.69 in gestational week 24. In our previous study of preeclampsia prediction in *early* pregnancy, we found an AUC of 0.68 and 0.67 for *preterm* and *term* preeclampsia, respectively^44^.

In the latest United States Preventive Services Task Force (USPSTF) recommendations, screening for preeclampsia with blood pressure measurements throughout pregnancy is emphasised^2^. Previous knowledge suggests that prehypertension, and blood pressure patterns throughout pregnancy, may be important for prediction of preeclampsia^21–24^. We have not found any longitudinal predictive studies of preeclampsia restricted to nulliparous women. A previous study using maternal characteristics and serial blood pressure measurements in a multivariable model (all parities), demonstrated improved prediction of preeclampsia from gestational week 28 and onwards (AUC 0.79 [95% CI 0.77–0.82] in gestational week 24, to 0.88 [95% CI 0.86–0.90] in week 36)^18^. Our findings in nulliparous women displayed a similar improvement of preeclampsia prediction, although including additional longitudinal predictors to blood pressure measurements. Prediction of both *preterm* and *term* preeclampsia can be improved by using maternal factors and serial MAP, compared to MAP from only one trimester^45^.

The USPSTF’s preeclampsia screening recommendations further emphasize the need for high quality studies and models using parameters available in routine care^2^. Early pregnancy clinical risk factors are well established^46^. However, there is also a body of evidence supporting the importance of incorporating pregnancy trajectories of clinical examinations. Examples of factors are: weight gain during pregnancy^27–29^, especially for the risk of *term* preeclampsia^27^, symphysis-fundal height, since fetal growth restriction is strongly associated with preeclampsia^1^, hyperglycemia and gestational diabetes^30,31^. Isolated gestational proteinuria is associated with preeclampsia^32,33^, and so is increased hemoglobin concentration in second trimester (proxy for plasma volume expansion)^26,47^. To our knowledge, no previous study has addressed the predictive capacity of these variables in a multivariable model.

*Late-onset* preeclampsia (delivery ≥ 34 weeks’ gestation), comprise the majority of preeclampsia cases, and is 3–7 times more common than the generally more severe *early-onset* preeclampsia^48^. *Late-onset* preeclampsia is nevertheless associated with fetal growth restriction, perinatal morbidities and deaths, and maternal eclamptic seizures^48,49^. Irrespective of using MAP in first, second, or third trimester, the predictive capacity for *term* preeclampsia is consistently lower compared to *preterm* preeclampsia^15,16,50,51^. This is accordance with our results, but we found a major improvement for prediction of *term* preeclampsia by adding serial information. Close monitoring of the high risk group in third trimester enables diagnosis of hypertensive disorders at an early stage, and improve perinatal outcomes by both optimized treatment, and selection of appropriate time, place and method of delivery^7^. Compared to expectant management, planned delivery from 34 to 37 weeks’ gestation is associated with reduced maternal morbidity in women with mild hypertensive disease without adverse neonatal outcomes among term pregnancies^7,52^.

Biophysical examinations and numerous biomarkers have been proposed in second and third trimester prediction of preeclampsia. Improved prediction can be reached by adding second and/or third trimester uterine artery ultrasound examinations, and some biomarkers have displayed fairly good risk discrimination when used in second or third trimester, especially in combination with maternal characteristics^34,53–57^.

### Strengths and limitations

The strengths of this study include our linkage of multiple population registers including electronic medical records during antenatal, delivery and postpartum care and inpatient/outpatient visit records. The comprehensive range of prospectively measures in a standardized way, including serial medical examinations throughout pregnancy, compose a distinct strength. There is generally a minimal level of missing values and the data is population-based on a large population increasing the likelihood of accurate prediction and allowed us to study preeclampsia subtypes.

The analytical approach efficiently exploits the information jointly contained in the baseline and longitudinal predictors. The shared-effects joint longitudinal model utilizes all of the longitudinal measurements on each woman, irrespective of timing and number of observations, without using imputed data. These updated predictions are potentially more precise than those from traditional prognostic methods where routinely information is not evaluated in a multivariable or longitudinal fashion.

Several limitations should be noted. Analysis of secondary data did not make it possible to assess the potential misclassification of the mainly self-reported maternal characteristics. This would however probably reflect the outcomes of the model in the clinical setting. The medical examinations are performed according to guidelines by trained midwives in antenatal care. Blood pressure measurements may though have been rounded to closest 5 or 10 when registered in the medical record, influencing the specificity of the prediction.

The use of ICD-10 codes for preeclampsia instead of data from medical records can introduce misclassification bias. In order to improve the accuracy of the diagnosis, one diagnosis in in-patient or two diagnoses in outpatient care was required. The Swedish version of ICD-10 diagnoses defined preeclampsia with mandatory proteinuria during the study period, which is less sensitive but more specific compared to current international recommendations of the diagnosis^11^. Overall rates of preeclampsia in nulliparous women in our study were consistent with previous populations from western countries^36,37,53^.

### Perspective

By using a higher false positive rate cut off in week 36 than presented in this study, higher sensitivity for preeclampsia cases would be reached, and may be used for a broader definition of high-risk women. This could potentially also be a target for a two-stage screening with addition of biophysical or biochemical markers in the high-risk group. Using serial clinical data together with biophysical or biochemical markers is not well elucidated and has to be further studied regarding clinical performance and cost-effectiveness. In addition, to delineate which variables in our model that drives its predictive ability was not part of the scope of this study, but is of high importance and should be addressed in future research.

## Conclusion

By using routinely clinical information from first, second and third trimester in multivariable models with our statistical approach, identification of women who are at risk of developing both *preterm* and *term* preeclampsia can be improved and updated at each visit in antenatal care. This could be used to stratify antenatal care between women who require a more intensive monitoring from those with low risk of preeclampsia. Further studies to reach a high predictive accuracy with remained accessibility and affordability are needed.

## Supplementary Information

Supplementary Information.

## Data Availability

In the ethical approval of the study and in informed consent from the caregivers in Stockholm County Council we were given access to data to conduct the study but were not given permission to share data. However, statistical analysis code (STATA and R) is available on request from the corresponding author.
