# Supplementary material for: Routinely collected antenatal data for longitudinal prediction of preeclampsia in nulliparous women: a population-based study
Source: Sci Rep. 2021 Sep 9;11:17973. doi: 10.1038/s41598-021-97465-3 (PMC8429420; doi:10.1038/s41598-021-97465-3)
Supplement: Supplementary file 1 — Supplementary Information. [file 41598_2021_97465_MOESM1_ESM.pdf]

Data supplement

Appendix S1

This appendix describes in detail the features, estimation, and performance assessment of the predictive models reported in the paper.

Features of the predictive models

We developed predictive models for the probability of diagnosis of preeclampsia overall, preeclampsia with delivery before 37 weeks' gestation and preeclampsia with delivery from 37 weeks' gestation. Additional predictive models for the probability of preeclampsia before and from 37 weeks' gestation (irrespective of gestational length at delivery) were created. Each model included 20 baseline predictors (e.g. maternal characteristics and medical history) and two time-varying predictors that were not treated as continuous (plasma glucose: binary, if > 9 mmol/L, then positive from that date and onwards, and proteinuria that was categorical, if 1+ or >=2+, then treated as that from that date and onwards). The five continuous time-varying predictors (systolic blood pressure, diastolic blood pressure, hemoglobin, maternal weight and symphysis-fundal height) were included in the models by means of u-scores. For each time-varying predictor, a set of three u-scores captured the departure of each woman's trajectory from the non-preeclamptic population average trajectory with respect to three features: level, trend, and curvature.

Figure S1 illustrates these features with an example for systolic blood pressure, one of the time-varying predictors. The average trajectory of systolic blood pressure in the non-preeclamptic population is indicted by the solid curve, and that of a fictitious woman by a dashed curve. The observed measures of her systolic blood pressures are displayed as dots. The woman's trajectory has higher level, steeper trend, and more pronounced curvature, than the population average.

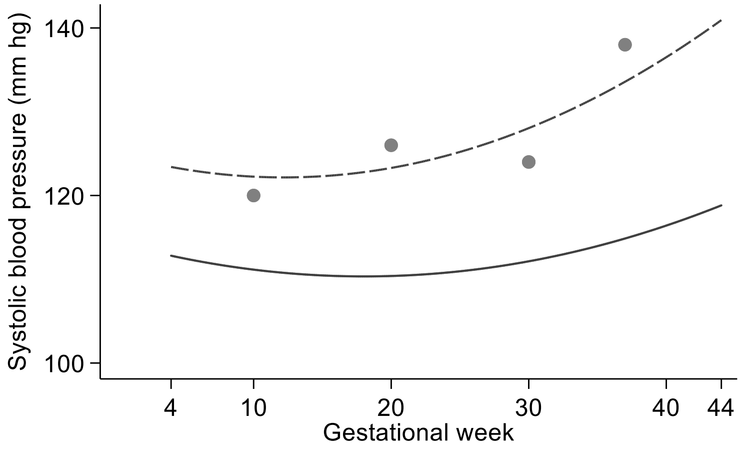

Figure S1. The non-preeclamptic population average trajectory of systolic blood pressure (solid curve), and the predicted trajectory (dashed curve) and the observed measures (dots) of a fictitious woman.

Estimation of the predictive models

We estimated the predictive models in three steps: first, we defined and estimated a mixed-effects model with data from the population of women without pre-eclampsia; second, we used the empirical best linear unbiased predictor to obtain the u-scores for level, trend, and curvature, for all women; third, we included the u-scores along with the other baseline predictors in logistic regression models. The following three subsections describe the three steps in detail.

First step: define and estimate the trajectory of time-varying predictors

After excluding all the women with pre-eclampsia, we estimated the following mixed-effect model,

$$W_{i,j} = (\alpha_0 + u_{i,0}) + (\alpha_1 + u_{i,1})\text{week}_{i,j} + (\alpha_2 + u_{i,2})\text{week}_{i,j}^2 + e_{i,j}$$

where the subscript i indicated the woman, the subscript j the visit, $W_{i,j}$ is the observed predictor $W_{i,j}$, $\text{week}_{i,j}$ the gestational week, $u_{i,0}$, $u_{i,1}$, $u_{i,2}$ the random effects, $e_{i,j}$ the residual term, and α_0, α_1, and α_2 the regression coefficients.

Although the above second-order polynomial model was an approximation to the actual trajectories of the different time-varying predictors, we deemed it adequate to capture their main features, namely level, trend, and curvature. The level represented the average of the predictor if this was constant over time. The level was α_0 for the population and $(\alpha_0 + u_{i,0})$ for the i-th woman. The trend represented the slope of the trajectory if this was linear. The trend was α_1 for the population and $(\alpha_1 + u_{i,1})$ for the i-th woman. Finally, the curvature represented the convexity of the trajectory as measured by its second derivative. The curvature was α_2 for the population and $(\alpha_2 + u_{i,2})$ for the i-th woman.

The random effects $u_{i,0}$, $u_{i,1}$, $u_{i,2}$ represented the departure of the i-th woman's trajectory from the non-preeclamptic population-average trajectory of the level, trend, and curvature, respectively. Figure S2 helps visualize their interpretation.

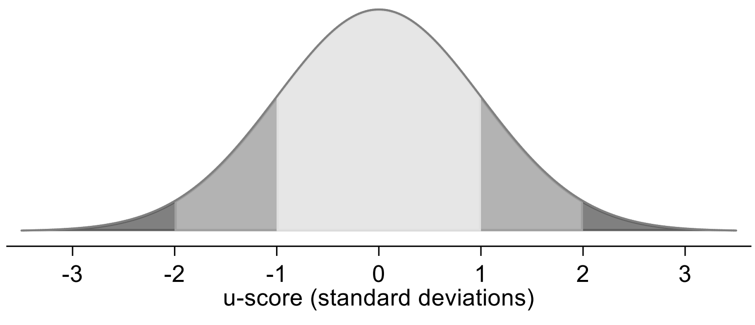

Figure S2. The distribution of the u-score in the non-preeclamptic population for any given feature (level, trend, and curvature) of a woman's trajectory. The x-axis indicates the standard deviations from the population average. When the u-score of a given feature and woman is equal to zero, that feature of that woman' trajectory is equal to population average. A positive (negative) u-score indicates that the feature is larger (smaller) than that of population.

The random vector $u_i = (u_{i,0}, u_{i,1}, u_{i,2})'$ was assumed to follow a multivariate normal distribution with mean equal to the three-dimensional vector of zeros and covariance matrix equal to

$$G = \begin{bmatrix} \gamma_{0,0} & \gamma_{0,1} & \gamma_{0,2} \\ \gamma_{0,1} & \gamma_{1,1} & \gamma_{1,2} \\ \gamma_{0,2} & \gamma_{1,2} & \gamma_{2,2} \end{bmatrix}$$

The variance parameters $\gamma_{0,0}$, $\gamma_{1,1}$, and $\gamma_{2,2}$ were constrained to be positive. The remaining covariance parameters $\gamma_{0,1}$, $\gamma_{0,2}$, and $\gamma_{1,2}$ were left unconstrained. The residual term $e_{i,j}$ in the mixed model was assumed to follow a zero-mean normal distribution with variance σ_e^2. The vector u_i and the residual $e_{i,j}$ were assumed independent of each other and of the gestational week. The parameters α and γ were estimated by maximizing the corresponding likelihood function (McCulloch et al 2008).

<u>Second step: obtain the u-scores</u>

For all the women in the sample, we obtained the u-scores with the empirical best linear unbiased predictor (EBLUP),

$$u_i = GZ_i'(Z_iGZ_i' + \sigma_e^2 I_i)^{-1}(w_i - Z_i\alpha)$$

where the vector w_i contained the measures of the time-varying predictor at each visit, Z_i indicated the matrix with the j-th row equal to $(1, \text{week}_{i,j}, \text{week}_{i,j}^2)$ for the j-th visit for the i-th woman, and I_i indicated the identity matrix. The number of rows of the matrices Z_i and I_i was equal to the number of visits for the i-th woman, which varied across women. The parameters α and γ contained in the above expression were replaced with the estimates obtained in the first step, as described in the previous section.

The EBLUP has desirable properties and an interesting interpretation (McCulloch et al 2008). To predict a given woman's trajectory, it optimally merges the information contained in the observations available for that woman and that contained in the sample of all women. More specifically, the EBLUP predicts the departure of her trajectory from the population-average trajectory by shrinking the observed residual $(w_i - Z_i\alpha)$ by a factor $GZ_i'(Z_iGZ_i' + \sigma_e^2 I_i)^{-1}$. When the shrinkage factor is large, the predicted trajectory is close the non-preeclamptic population-average trajectory. When shrinkage factor is small, the predicted trajectory is close to the woman's observed values. The level of shrinkage depends on two quantities: (1) the number of observations (visits) available, and (2) the relative magnitude of the variability between and within women. When the number of available observations is large and the trajectories vary substantially from woman to woman, little shrinkage takes place for that woman. Conversely, when the number of visits and the woman to woman variability are small, the shrinkage is considerable. Because the shrinkage depends on the number of observations available on each woman, it varies across women.

<u>Third step: predict the probability of pre-eclampsia</u>

For each binary outcome, (pre-eclampsia, preeclampsia with delivery before 37 weeks' gestation, preeclampsia with delivery from 37 weeks' gestation, diagnosis of preeclampsia before, and from 37 weeks' gestation), we estimated a generalized linear model with logit link and normal family distribution:

$$\text{logit } P(Y_i = 1) = \beta_0 + \beta_1 x_{i,1} + \cdots + \beta_p x_{i,p} + \beta_{p+1} u_{i,1} + \cdots + \beta_{i,p+q} u_{i,q}$$

where Y_i indicates the binary outcome of the i-th woman, $x_{i,j}$ the j-th of her p covariates, and $u_{i,j}$ the j-th of her q u-scores. The baseline predictors were: maternal age at baseline, region of birth, family situation, height, smoking 3 months before pregnancy and at first antenatal visit, previous miscarriage, infertility duration, infertility treatment, family history of preeclampsia and hypertension, chronic diseases (cardiovascular disease, endocrine disease, pre-excisting diabetes, thrombosis history, systemic lupus erythematosus (SLE), chronic hypertension, Mb Crohn/Ulcerative colitis, kidney disease and blood group . The time-varying predictors capillary glucose and proteinuria were treated as binary and categorical, respectively. The u-scores were calculated for systolic blood pressure, diastolic blood pressure, maternal weight, symphysis fundal height and haemoglobin level. The baseline predictors, capillary glucose, proteinuria and the u-scores entered the predictive models without any further selection.

On the estimation of the standard errors

The three-step approach described above implied that the standard errors for the coefficients β of the logistic regression calculated in Step 3 were possibly underestimated, as they did not take into account the uncertainty inherent in the estimates of the quantities obtained in Steps 1 and 2. Bootstrapping the full three-step process or maximizing the joint likelihood would provide correct standard errors. We performed neither of these alternative approaches, however, because standard errors, p-values, and confidence intervals, were inconsequential in any of the above steps. In addition, bootstrapping or maximizing a joint likelihood with our large sample was unfeasible with the computing resources available to us.

Assessment of the performance of the predictive models

The goodness of fit of the model as assessed with the Hosmer-Lemeshow test and its sensitivity and specificity were summarized by the area under the curve (AUC), and by sensitivity for 10% false positive rate.

We assessed the performance of the predictive models under five different scenarios: 1) included all available visits up to 24 fully gestational weeks (168 days), 2) all visits up to 28 gestational weeks (196 days), 3) all visits up to 32 gestational weeks (224 days), 4) all visits up to 34 gestational weeks (238 days) and 5) all visits up to 36 gestational weeks (252 days). The scenarios allowed evaluating the performance of the predictive models when the measures of the time-varying predictors at future visits are still unknown. At the 24th gestational week prediction, the u-scores for symphysis fundal height were unavailable, as this predictor is generally measured at later times during gestation.

The number of visits available on each women varied across the five scenarios, which meant that the number of rows of the vector w_i and of the matrices Z_i and I_i for the calculation of the u-scores in the second step also varied across scenarios. The parameters α, β, and γ, however, were constant across the scenarios, as these were estimated in the first and third step.

Table S1. Number of observations of time-varying predictive variables in antenatal care in nulliparous women

	Number of observations in antenatal care						
**Longitudinal predictive variables in antenatal care**		**Overall**		**Without preeclampsia**		**With preeclampsia**	
	Missing (N)	Median	10th, 90th percentile	Median	10th, 90th percentile	Median	10th, 90th percentile
**Visits to antenatal care**		11	8, 22	11	8, 22	12	7, 24
**Systolic blood pressure**, mmHg		10	7, 20	10	7, 20	11	7, 22
**Diastolic blood pressure**, mmHg	2	10	7, 20	10	7, 20	11	7, 22
**Weight,** kg	1 092	6	2, 12	6	2, 12	6	2, 13
**Hemoglobin (Hb)**, g/L	9	6	3, 12	6	3, 12	5	3, 12
**Symphysis-fundal height**, cm	252	8	5, 16	8	5, 16	7	4, 14
**Capillary glucose**, mmol/L	145	5	2, 10	5	2, 10	5	2, 10
**Proteinuria**, dipstick 0-4	1 316	4	1, 10	4	1, 10	6	2, 16

Table S2. Mean U-scores for level, trend and curvature trajectories of the time-varying predictive variables in antenatal care, by preeclampsia, in nulliparous women

	Mean U-scores of time-varying Predictors*		
**Time-varying predictors**	**Without preeclampsia n= 56 323**	**With preeclampsia n=2 576**	**P-Value**
**Systolic blood pressure**			
Level	.001017	-.4682232	<0.001
Trend	-.0000319	.0151942	<0.001
Curvature	-.0155968	9.660262	<0.001
**Diastolic blood pressure**			
Level	-.0002188	-.4527302	<0.001
Trend	-2.85e-06	.0147889	<0.001
Curvature	-.0049667	8.066177	<0.001
**Haemoglobin (Hb)**			
Level	-.0024374	.1954536	<0.001
Trend	.0000484	-.0045065	<0.001
Curvature	.014297	-.2157052	0.35
**Weight (kg)**			
Level	.0015437	-.1324119	<0.001
Trend	-.0000333	.0042093	<0.001
Curvature	-.0410137	5.102106	<0.001
**Symphysis-fundal height**			
Level	.0007745	-.0232342	0.052
Trend	-.0000137	.0005151	0.005
Curvature	-.0118639	.3410827	0.071

* For each time-varying predictor, a set of three u-scores captured the departure of each woman's trajectory from the non-preeclamptic population average trajectory with respect to three features: level, trend, and curvature. A positive or negative u-score indicates that the feature is larger or smaller than that of the non-preeclamptic population.

Table S3. Performance of the prediction models for diagnosis of preeclampsia < 37 weeks' and ≥ 37 weeks' gestation (irrespective of gestational age at delivery) at different gestational ages

	Diagnosis of preeclampsia < 37 weeks				Diagnosis of preeclampsia ≥ 37 weeks			
**Gestational age of prediction* (weeks)**	**AUC†**	**(95% CI)**	**Sensitivity for 10% FPR‡**	**(95% CI)**	**AUC†**	**(95% CI)**	**Sensitivity for 10% FPR‡**	**(95% CI)**
**24**	**0.74**	(0.70-0.79)	**37.5**	(29.8-45.7)	**0.63**	(0.60-0.66)	**21.8**	(17.3-26.9)
**28**	**0.78**	(0.76-0.80)	**41.2**	(37.1-45.3)	**0.64**	(0.63-0.66)	**21.7**	(19.6-23.9)
**32**	**0.83**	(0.81-0.85)	**56.5**	(52.1-60.9)	**0.67**	(0.66-0.69)	**25.4**	(23.2-27.7)
**34**	**0.86**	(0.83-0.88)	**64.1**	(59.1-69.0)	**0.71**	(0.70-0.73)	**31.3**	(28.9-33.7)
**36**	**0.88**	(0.85-0.91)	**69.6**	(61.2-77.1)	**0.77**	(0.75-0.78)	**40.4**	(37.9-43.0)

* The model is composed of the predictive variables collected at first antenatal visit, the time-varying predictors plasma glucose and proteinuria, and the u-scores of level, trend and curvature for each of the time-varying predictors systolic and diastolic blood pressure, haemoglobin, maternal weigh and symphysis fundal height up until the gestational week of prediction (24, 28, 32, 34 and 36).

† AUC: Area under receiver operating characteristic curve

‡ FPR: False positive rate

Table S4. Sensitivity analysis excluding women with aspirin treatment during pregnancy. Performance of the predictive models for diagnosis of preeclampsia at different gestational ages during pregnancy (N=58 276)

		Preeclampsia		
**Gestational age of prediction* (weeks)**	**AUC†**	**(95% CI)**	**Sensitivity for 10% FPR‡**	**(95% CI)**
**24**	**0.68**	(0.66- 0.71)	**28.7**	(24.5- 33.0)
**28**	**0.70**	(0.69-0.71)	**29.9**	(27.9-31.9)
**32**	**0.73**	(0.72-0.75)	**35.2**	(33.0-37.3)
**34**	**0.77**	(0.75-0.78)	**41.0**	(38.8-43.3)
**36**	**0.80**	(0.78-0.81)	**46.4**	(44.0-48.9)

* The model is composed of the predictive variables collected at first antenatal visit, the time-varying predictors plasma glucose and proteinuria, and the u-scores of level, trend and curvature for each of the time-varying predictors systolic and diastolic blood pressure, haemoglobin, maternal weigh and symphysis fundal height up until the gestational week of prediction (24, 28, 32, 34 and 36).

† AUC: Area under receiver operating characteristic curve

‡ FPR: False positive rate

Table S5: Coefficients of the parameters of the predictive model

Predictive variables	Diagnosis of Preeclampsia overall	Diagnosis of preeclampsia < gw 37	Diagnosis of preeclampsia ≥ gw 37
**Constant**	-0.8929	-2.8400	-2.2478
**Maternal age at first antenatal visit**	0.0163	0.0405	-0.0009
**Height**	-0.0277	-0.0315	-0.0153
**Previous miscarriage**	-0.0025	-0.0011	-0.0045
**Infertility duration**	0.0033	0.0585	-0.0336
**Infertility treatment**			
Ovary stimulation	0.1643	0.0442	0.0785
IVF	0.1153	-0.1235	0.3040
**Family situation**			
Living together with partner	ref	ref	ref
Single	0.1141	0.4487	0.0079
Other	0.0110	0.0628	-0.0316
**Region of birth**			
Sweden	ref	ref	ref
Nordic countries (except of Sweden)	-0.1436	-0.3545	-0.0551
Europe (except of Nordic countries)	-0.3207	-0.3189	-0.2687
Africa	0.4370	0.4796	0.1777
North America	-0.1677	-0.1154	-0.2676
South America	-0.2766	0.2741	-0.3323
Asia	-0.2035	0.0361	-0.2782
Oceania	-1.8542	-	-0.3087
**Smoking 3 months before pregnancy**			
<10	-0.1539	-0.2379	-0.0643
≥10	-0.3721	-0.4369	-0.2750
**Smoking at first antenatal visit**			
<10	-0.1874	0.3583	-0.3033
≥10	0.3584	0.5514	0.2250
**Family history of preeclampsia**	0.2449	0.2635	-0.0656
**Family history of hypertension**	-0.0010	0.0077	-0.0132
**Cardiovascular disease**	0.0503	-0.4509	0.1086
**Endocrine disease**	0.0026	-0.3339	0.1501
**Pre-existing diabetes**	-0.0228	1.0462	-0.7219
**Thrombosis**			
**SLE** n (%)	-	-	-
**Chronic hypertension**	-0.2917	-0.6951	-0.0636
**Mb Crohn/Ulcerous colitis**	-0.4297	-0.4830	-0.4422
**Chronic kidney disease**	0.0245	0.3081	0.0458
**Blood group**			
0	ref	ref	ref
A	0.0453	0.0741	0.0104
AB	0.0180	0.2224	-0.0181

B	0.0684	0.1646	-0.0038
**Systolic blood pressure**			
Level	2.3122	1.7770	1.5400
Trend	108.8857	59.9576	84.1703
Curvature	0.0392	0.0334	0.0290
**Diastolic blood pressure**			
Level	4.6273	7.2351	1.8809
Trend	190.4049	270.3426	92.0414
Curvature	0.0967	0.1421	0.0459
**Hemoglobin (Hb)**			
Level	0.0798	0.5894	-0.2069
Trend	1.1189	16.0010	-7.4970
Curvature	-0.0008	0.0129	-0.0067
**Weight (kg)**			
Level	1.3098	1.4453	0.8698
Trend	94.1729	98.9102	61.1168
Curvature	0.0057	0.0037	0.0073
**Symphysis-fundal height**			
Level	-4.6849	-11.5770	0.9998
Trend	-156.4996	-398.3259	43.3697
Curvature	-0.1442	-0.3375	0.0168
**Capillary glucose**	-0.0526	0.0035	-0.0350
**Protein in urine**	1.5585	1.3116	1.2644

Table S6. Parameters of the mixed effect model

**Predictive variables**	**Systolic blood pressure**	**Diastolic blood pressure**	**Weight**	**Hb**	**Symphysis-fundal**
**Level**	114.6837	72.7151	61.6429	143.4706	-12.0752
**Trend (time_1)**	-0.4742	-0.7969	0.2947	-2.0566	1.8301
**Curvature (time_2)**	0.0129	0.0199	0.0044	0.0412	-0.0156
**log(gamma11)**	0.1272	-0.1454	-0.8851	0.3829	-0.2697
**log(gamma22)**	-3.7747	-4.0120	-4.7969	-3.4735	-4.4372
**log(gamma33)**	2.7192	2.4180	2.4933	2.7959	2.4914
**arctan(gamma12)**	-2.1036	-2.0763	-1.5960	-2.1504	-2.8509
**arctan(gamma13)**	-1.1099	-1.2099	-0.4048	-1.2888	-2.7268
**arctan(gamma23)**	0.8912	0.9801	0.3597	1.0066	2.1539
**log(sigma_e)**	1.8553	1.5810	-0.0586	1.7188	-0.6552
